# Metabolomic approach to search for fungal resistant forms of Aegilops tauschii Coss. from the VIR collection

**DOI:** 10.18699/VJ20.618

**Published:** 2020-05

**Authors:** T.V. Shelenga, L.L. Malyshev, Yu.A. Kerv, T.V. Diubenko, A.V. Konarev, V.I. Horeva, M.K. Belousova, M.A. Kolesova, N.N. Chikida

**Affiliations:** Federal Research Center the N.I. Vavilov All-Russian Institute of Plant Genetic Resources (VIR), St. Petersburg, Russia; Federal Research Center the N.I. Vavilov All-Russian Institute of Plant Genetic Resources (VIR), St. Petersburg, Russia; Federal Research Center the N.I. Vavilov All-Russian Institute of Plant Genetic Resources (VIR), St. Petersburg, Russia; Federal Research Center the N.I. Vavilov All-Russian Institute of Plant Genetic Resources (VIR), St. Petersburg, Russia; Federal Research Center the N.I. Vavilov All-Russian Institute of Plant Genetic Resources (VIR), St. Petersburg, Russia; Federal Research Center the N.I. Vavilov All-Russian Institute of Plant Genetic Resources (VIR), St. Petersburg, Russia; Federal Research Center the N.I. Vavilov All-Russian Institute of Plant Genetic Resources (VIR), St. Petersburg, Russia; Federal Research Center the N.I. Vavilov All-Russian Institute of Plant Genetic Resources (VIR), St. Petersburg, Russia; Federal Research Center the N.I. Vavilov All-Russian Institute of Plant Genetic Resources (VIR), St. Petersburg, Russia

**Keywords:** Aegilops tauschii Coss, metabolomic approach, disease resistance, fungal pathogens, Aegilops tauschii Coss, метаболомный подход, устойчивость к болезням, грибные патогены

## Abstract

Broadening of the genetic diversity of donors of resistance to biotic environmental factors is a challenging problem concerning Triticum L., which can be solved by using wild relatives of wheat, in particular, Aegilops tauschii Coss., in breeding programs. This species, believed to be the donor of D genome of common wheat (T. aestivum L.), is a source of some traits important for breeding. This greatly facilitates the possibility of crossing Ae. tauschii with common wheat. Aegilops L. species are donors of effective genes for resistance to fungal diseases in wheat. For instance, genes that determine resistance to rust agents in common wheat were successfully introgressed from Ae. tauschii into the genome of T. aestivum L. The aim of our study was to identify differences in metabolomic profiles of Ae. tauschii forms (genotypes), resistant or susceptible to such fungal pathogens as Puccinia triticina f. sp. tritici and Erysiphe graminis f. sp. tritici. These indicators may be used as biochemical markers of resistance. A comparative analysis of groups of Ae. tauschii accessions showed that metabolomic profiles of the forms with or without resistance to fungal pathogens differed significantly in the contents of nonproteinogenic amino acids, polyols, phytosterols, acylglycerols, mono- and oligosaccharides, glycosides, phenolic compounds (hydroquinone, kempferol), etc. This fact was consistent with the previously obtained data on the relationship between Fusarium resistance in oats (Avena sativa L.) and certain components of the metabolomic profile, such as acylglycerols, nonproteinogenic amino acids, galactinol, etc. Thus, our studies once again confirmed the possibility and effectiveness of the use of metabolomic analysis for screening the genetic diversity of accessions in the VIR collection, of Ae. tauschii in particular, in order to identify forms with a set of compounds in their metabolomic profile, which characterize them as resistant. Ae. tauschii accessions with a high content of pipecolic acids, acylglycerols, galactinol, stigmasterol, glycerol, azelaic and pyrogallic acids, campesterol, hydroquinone, etc., can be used for creating wheat and triticale cultivars with high resistance to fungal pathogens causing powdery mildew, brown rust, and yellow rust.

## Abstract (ru)

Расширение генетического разнообразия доноров устойчивости Triticum L. к биотическим факторам среды – актуальная задача, которую возможно решить благодаря использованию в селекционных программах дикорастущих родичей пшеницы, в частности Aegilops tauschii Coss. По существующим представлениям, последний – донор генома D мягкой пшеницы T. aestivum L. и носитель ряда ценных селекционных признаков. Это значительно облегчает скрещивание Ae. tauschii с мягкой пшеницей. Виды рода Aegilops L. являются донорами эффективных генов устойчивости пшеницы к грибным болезням. Так, от видов Aе. tauschii в геном T. aestivum L. успешно интрогрессированы гены, детерминирующие устойчивость мягкой пшеницы к возбудителям ржавчины. Целью нашего исследования было выявление различий по метаболомным профилям форм (генотипов) Ae. tauschii, устойчивых и неустойчивых к грибным патогенам (Puccinia triticina f. sp. tritici и Erysiphe graminis f. sp. tritici ), что позволит использовать такие показатели в качестве биохимических маркеров устойчивости. Сравнительный анализ групп образцов Ae. tauschii показал, что метаболомные профили устойчивых и неустойчивых к грибным патогенам форм достоверно различаются между собой по содержанию небелковых аминокислот, многоатомных спиртов, фитостеролов, ацилглицеролов, моно- и олигосахаридов, гликозидов, фенольных соединений (гидрохинона, кемпферола) и др. Последнее подтверждается ранее полученными нами данными о связи устойчивости овса (Avena sativa L.) к возбудителям фузариоза с определенными компонентами их метаболомного профиля: ацилглицеролами, небелковыми аминокислотами, галактинолом и др. Таким образом, наши исследования еще раз подтвердили возможность и эффективность применения метаболомного анализа для скрининга генетического разнообразия образцов коллекции ВИР, в частности Ae. tauschii, с целью выделения форм, имеющих в метаболомном профиле набор соединений, характеризующих их как устойчивые. К таковым относятся образцы Ae. tauschii с высоким содержанием пипеколиновых кислот, ацилглицеролов, галактинола, стигмастерола, глицерола, азелаиновой и пирогалловой кислот, кампестерола, гидрохинона и др., которые могут быть использованы для создания сортов пшеницы и тритикале, высокоустойчивых к грибным патогенам – возбудителям мучнистой росы, бурой и желтой ржавчины.

## Introduction

Wheat (Triticum L.) is one of the most significant crops in the world, including the Russian Federation. For the majority of the world’s population, it is one of the staple foods. The yield and quality of wheat largely depend on the resistance of cultivars to environmental stress factors, including fungal diseases. Most of the cultivated foreign and domestic cultivars are susceptible to diseases caused by agents of stem rust (Puccinia graminis Pers. f. sp. tritici Erikss. et Henn), brown rust (Puccinia recondita Rob. ex Desm f. sp. triticina Eriks.), mildew (Blumeria graminis (DC.) Speer f. sp. tritici Marchal.) and septoria leaf blotch (Mycosphaerella graminicola (Fuckel) J. Schroet. (=Septoria tritici); Phaeosphaeria nodorum (E. Muell.) Hedjar. (=Leptosphaeria nodorum E. Muell., =Septoria nodorum (Berk.)). Crop losses can reach up to 40 % (Afanasenko, 2010; Kolomiets et al., 2017). The creation of wheat cultivars resistant to the most harmful fungal pathogens is one of the effective ways to combat them.

Wild relatives of cultivated forms of wheat, rye, barley, oats, etc. serve as an inexhaustible source of resistance genes for creating cultivars combining high yields and resistance to environmental factors. Evolutionary, Aegilops L. species are close to those of the genus Triticum L. (Dorofeev, 1971; Migushova, 1975; Konarev, 1980; Liu et al., 2015; Arora et al., 2017). However, the donors used in breeding programs, in most cases are characterized by the same resistance genes. With time, it leads to the appearance of “adapted” forms of pathogens that infect cultivars previously considered to be resistant. Many known resistance genes from Ae. tauschii Coss. are not used in practice to improve wheat cultivars, as their protective effect is considered low (Pretorius, 1997; Kolmer, Anderson, 2011). Expansion of the genetic diversity of donors of resistance to wheat fungal diseases will help breeders solve this problem, and the global collection of wheat wild relatives at the N.I. Vavilov All-Russian Research Institute of Plant Industry (VIR) plays a crucial role in this task (Vavilov, 1919).

The VIR collection of the genus Aegilops L. contains over 5,000 accessions of various ecological and geographical origins, and it includes thirteen diploid, ten tetraploid, and five hexaploid species. Since 1956, species possessing complex immunity to fungal diseases have been identified in the collection: diploid Ae. mutica Boiss., Ae. speltoides Tausch., Ae. aucheri Boiss., Ae. bicornis (Forsk.) Jaub. et Spach., Ae. comosa Sibth. & Sm., Ae. uniaristata Vis., Ae. heldreichii Hozm.; tetraploid Ae. ovata L., Ae. triaristata Wild., Ae. ventricosa Tausch., Ae. variabilis Eig. Such a diversity of genetic material makes it possible to select appropriate genotypes for the subsequent production of wheat cultivars with improved biological characteristics. Ae. tauschii is a carrier of D genome, which is close to the polyploid wheat genome, and this fact greatly facilitates the crossing of Ae. tauschii with common wheat when transmitting effective disease resistance genes (Dobrotvorskaia et al., 2017). Besides, flour of such cultivars has high baking quality (Semenova et al., 1973). By now, most of the effective genes that determine resistance to rust and wheat spot blotch agents have been successfully introgressed into the genome of T. aestivum L. (McIntosh et al., 1995; Mujeeb-Kazi et al., 2001; Yang et al., 2003; Adonina et al., 2012).

Recently, nonspecific metabolomic analysis has found wide application for plant species phenotyping and resistance studies, which provides a unique opportunity to scan a wide range of compounds that make up the metabolomic profile in the source material and give an objective assessment (using metabolomic markers) of the plant’s response to environmental factors (Konarev et al., 2015). This approach is increasingly used to identify individual metabolites or their groups that can characterize the protective status of the studied object, which makes it possible to identify accessions resistant to environmental stressors (Taji et al., 2006; Chakraborty, Newton, 2011; Valitova et al., 2016; Loskutov et al., 2017). Currently, metabolomic profiles of various crops from the VIR collection are investigated. Wild and cultivated forms of oats resistant and susceptible to Fusarium were studied, and significant differences among them were shown for a number of compounds (acylglycerols, nonproteinogenic amino acids, galactinol, etc.) (Loskutov et al., 2017, 2019). Total screening of wild forms of various crops will make it possible to shape a model “metabolomic profile of a resistant cultivar”.

The aim of this study was the identification of metabolomic markers to be used in screening the genetic diversity of wild relatives for the forms with effective resistance genes, which can be efficiently introduced into the common wheat genome for the use in breeding programs. The research objectives included the study of metabolomic profiles of Ae. tauschii accessions with and without resistance to leaf rust and powdery mildew pathogens, in order to detect metabolites marking the susceptibility of Ae. tauschii to fungal pathogens. The results of the study can shorten and optimize the selection of source material when creating wheat cultivars highly resistant to fungal pathogens.

## Materials and methods

Fifty Ae. tauschii accessions from the VIR collection (see the Table), grown at the Dagestan Experimental Station of VIR (DES VIR) in 2017 and harvested at full ripeness, were used as the material for the study. The sample was composed from the main of Ae. tauschii botanical varieties in the VIR collection, taking into account the most complete ecogeographical representation.

**Table 1.**
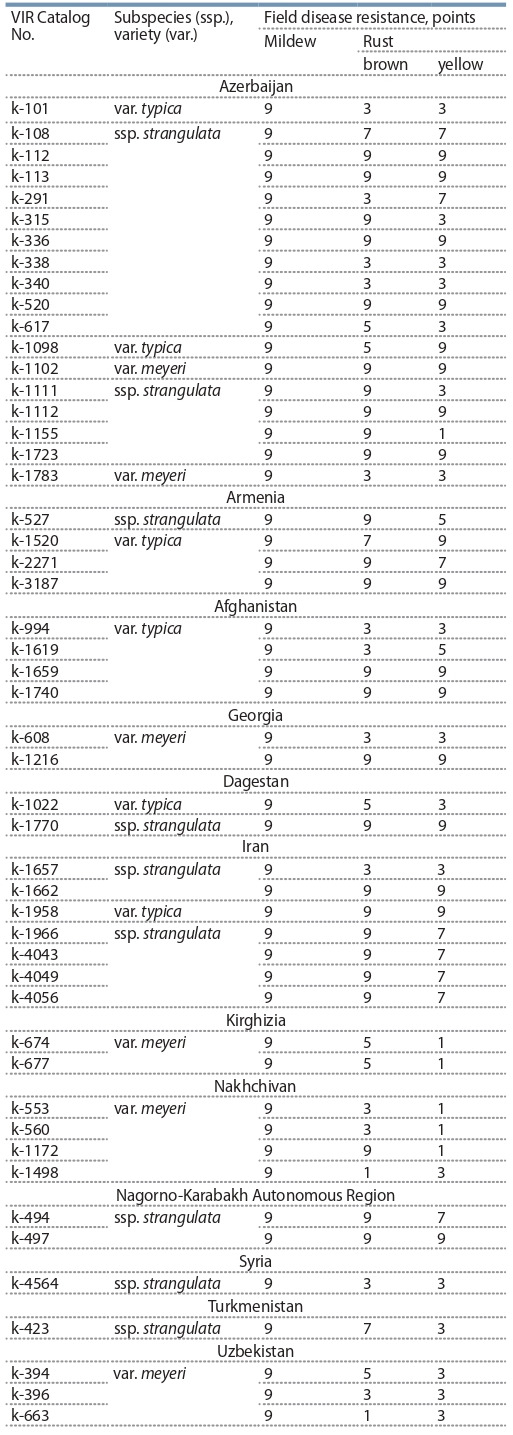
Leaf disease resistance in Ae. tauschii Coss. accessions

Field studies of Ae. tauschii accessions were conducted at DES VIR on irrigated 1-^2^ plots according to the method accepted in VIR (Merezhko et al., 1999). During the growing season, the air temperature averaged +20.4 °C, the amount of precipitation was 15.4–16.3 mm, and the total of active temperatures amounted to 3400–4500 °С. Field assessment of the infection of Ae. tauschii accessions by fungal pathogens was carried out in fields of DES VIR, and the laboratory evaluation of the degree of infection was carried out at the VIR Department of Genetics in compliance with methods employed at VIR (Tyryshkin et al., 2004). Resistance was determined on a 9-point scale, where 9 means the absence of disease symptoms or presence of small necrotic spots; 7, microscopic pustules surrounded by necrotic zone; 5, small pustules surrounded by a wide necrotic zone; 3, medium-size pustules surrounded by chlorotic tissue; and 1, large pustules forming continuous lesion zones. Plants scoring 9–7 were classified as resistant, and those with 5–1 points as susceptible. Plants were examined at 5-day intervals throughout the growing season. For each accession, an integral score was derived from the highest infection point (see the Table).

Metabolomic profiles of grain from Ae. tauschii accessions were studied at the Department of Biochemistry and Molecular Biology of VIR (in 5 biological and 3 analytical replications) (Loskutov et al., 2017). The grains were cleaned of glumes and ground; 50 mg of the flour of an accession were homogenized with 500 μL of methanol, and the sample was kept at 5–6 °C for 30 days. A 100-μL portion of the extract was evaporated to dryness using a Labconco CentriVap Concentrator (USA). The dry residue was silylated using bis(trimethylsilyl)trifluoroacetamide at 100 °C for 40 minutes. The separation of trimethylsilyl ethers of metabolites was carried out using an HP-5MS 5 % phenyl–95 % methylpolysiloxane capillary column (30.0 m, 250.00 μm, 0.25 μm) on an Agilent 6850 gas chromatograph with an Agilent 5975B VL quadrupole mass selective detector MSD (Agilent Technologies, USA). The analysis was performed at 1.5 mL/min inert gas flow rate through a column. The column was heated from +70 up to +320 °C at 4 °C/min heating rate. The temperature of the mass spectrometer’s detector was +250 °C, and that of the injector was +300 °C. The injected sample volume was 1.2 μL. Pyridine solution of tricosan (1 μg/μL) served as the internal standard.

The results were processed using UniChrom and AMDIS software. The peaks were identified using the NIST 2010 mass spectra library and as well as libraries of the St. Petersburg University Research Park and of the V.L. Komarov Botanical Institute of the Russian Academy of Sciences (Puzanskiy et al., 2015). The biochemical parameter values are given in ppm (μg/g).

Statistical data were evaluated with the Statistica 7.0 software package. The initial set of characters was screened by the method of one-way analysis of variance to identify the characters (metabolites) whose contents reliably discriminated Ae. tauschii accessions resistant and susceptible to the studied pathogens. The discriminant analysis was used to assess informativeness of resistance characters (metabolites) of Ae. tauschii accessions.

## Results

Metabolomic profiles of caryopses of Ae. tauschii accessions with and without resistance to fungal pathogens differed in several indicators. Higher contents of organic acids were observed in the resistant forms of Ae. tauschii (1190 ppm) compared with the susceptible ones (1090 ppm). The dominating organic acids in the metabolomic profile of Ae. tauschii caryopses were malic and methylmalonic acids (203 vs. 164 and 168 vs. 153 ppm, respectively). The caryopses of resistant and susceptible forms were found to contain, respectively, the following contents of organic acids (ppm): 102 and 79 galacturonic acid, 78 and 76 lactic, 43 and 48 gulonic, 25 and 44 gluconic, 30 and 47 azelaic, 32 and 38 succinic, 28 and 27 oxalic, 42.8 and 41.0 fumaric, 19.9 and 13.0 ribonic, and 11.5 and 10.0 glyceric. The sum of minor acids with concentrations no higher than 10 ppm each was 24.0 ppm in both resistant and susceptible forms of Ae. tauschii. All metabolic reactions in plants occur with the participation of phosphoric acid and its derivatives, which explains its rather high content in the studied accessions (320.2 in resistant and 277.0 ppm in susceptible forms), the amounts of methylphosphate being 64.0 and 56.0 ppm, respectively.

Three nonproteinogenic amino acids constituted 60 % of free amino acids in resistant and susceptible Ae. tauschii accessions: 3-hydroxypipecolic (137.2 and 116.6), pipecolic (0.5 and 0.3), and 5-hydroxypipecolic (0.7 and 0.7 ppm, respectively). The remaining amino acids were represented by essential (valine, isoleucine, threonine, phenylalanine, and tryptophan) and non-essential ones (α-alanine, glycine, serine, proline, hydroxyproline, asparagine, glutamine, tyrosine, aspartic acid, and glutamic acid). The dominating ones were asparagine (18.7 and 13.4 ppm), valine (15.6 and 16.0), α-alanine (10.6 and 13.1), glutamine (12.3 and 9.0) and glutamic acid (5.7 and 5.5 ppm), respectively. The amounts of the remaining amino acids did not exceed 4.0 ppm. The sums of free amino acids (except nonproteinogenic) did not differ significantly between the studied forms of Aegilops resistant and susceptible to fungal pathogens: 88.4 and 85.3 ppm, respectively.

Higher concentrations of polyols and phytosterols were recorded in the susceptible forms of Ae. tauschii (341.8 and 336.5 ppm, respectively). Glycerol, xylitol, dulcitol, myo-inositol and inositol derivatives (59.5, 84.4, 70.4, 23.4, 9.6 ppm, respectively) prevailed in caryopses of the susceptible forms, while galactinol (92.5 ppm) dominated in the resistant ones. Phytosterols were mainly represented by sitosterol, stigmasterol, and campesterol; their contents were 219.8, 85.8, and 30.9 ppm in the susceptible forms, and 160.3, 44.5, and 22.7 ppm in the resistant ones, respectively.

The dominating fatty acids in caryopses of both resistant and susceptible forms of Ae. tauschii were palmitic (584.7 and 602.0), stearic (173.5 and 187.0), oleic (493.9 and 520.0), and linoleic (1515.0 and 1545.0 ppm), respectively. No significant difference in the contents of individual fatty acids or their total values (2943.3 and 3064.0 ppm) was noted.

The caryopses of resistant forms of Ae. tauschii were found to have higher contents of acylglycerols (954.0 vs. 745.6 ppm), mainly due to diacylglycerol (DAG) amounting to 631.0 and 464.0 ppm, respectively. The amounts of monoacylglycerols, i. e., MAG-2 C18:3 and MAG-1 C18:1, were also higher in resistant forms: 188.0 vs. 152.7, 54.0 vs. 34.2 ppm. Оn the contrary, the content of MAG-1 C16:0 was higher in the susceptible forms: 94.7 vs. 81.0 ppm in resistant.

Monosugars in Ae. tauschii caryopses were represented mainly by hexoses (over 80 %). These values were higher (1164.8 ppm) for resistant forms of Aegilops than for susceptible ones (1026.7 ppm). Of the hexoses, glucose (819.8) was predominant in resistant forms, while in the susceptible ones these were glucose (455.4) and fructose (318.1 ppm). The total pentose contents (ribose and xylose) did not exceed 40 ppm. The contents of glycerol-3 phosphate did not differ significantly between resistant and susceptible forms of Aegilops. They amounted to 42.8 and 37.4 ppm, respectively.

Oligosaccharides in caryopses of resistant and susceptible forms of Ae. tauschii amounted to 13480.6 and 14920.0 ppm, respectively. They were mainly represented by sucrose and raffinose. The content of sucrose was higher in susceptible forms (11604.9), while that of raffinose was higher (4882.0 ppm) in resistant ones.

Derivative sugars, identified as methyl-D-galactopyranoside, were found in both resistant and susceptible forms of Ae. tauschii (169.2 and 84.4 ppm, respectively).

In the group of phenolic compounds, the contents of hydroquinone (93.6 in resistant and 74.0 ppm in susceptible accessions) were found to be the highest. The values for kempferol; pyrogallic, 2,3-dihydroxybenzoic, salicylic, and caffeic acids; and α-tocopherol were 29.1, 1.5, 0.2, 0.3, 0.4, 0.4 ppm in resistant and 14.0, 1.3, 0.2, 0.2, 0.4 and 0.3 ppm in susceptible forms.

The above data reflect the activity of metabolic processes in Ae. tauschii caryopses. This activity characterizes primary and secondary metabolism, i. e., exchange of nitrogen-containing compounds, including amino acids; the Krebs cycle; carbohydrate metabolism; glycolysis; pentose phosphate cycle; exchange of signal (inositol) compounds; shikimate and glyoxylate pathways; etc.

Statistical processing of the data showed that the metabolome of susceptible forms of Ae. tauschii differs with varying degrees of significance from that of resistant ones in a number of indicators. Resistant forms of Ae. tauschii were divided into three groups: the first comprised accessions resistant to all the pathogens tested, the second included those resistant to only brown and yellow leaf rust, while the third included mildew resistant accessions. The metabolomes of all resistant forms differed with a high degree of confidence ( p = 0.05) from those of susceptible ones in the contents of nonproteinogenic amino acids (pipecolic and 3-hydroxypipecolic), glycerol, ononitol, galactinol, sitosterol, stigmasterol, behenic acid, DAG, MAG-1 C16:1, ribose, sorbose, sucrose, maltose, and hydroquinone; and with confidence levels 0.1 > p > 0.05 for fumaric, galacturonic, gluconic acids, threonine, dulcitol, mannitol, chiro- and myo-inositol, pelargonic and arachinic fatty acids, MAG-1 C18:1, and fructose. At p = 0.05, metabolomes of accessions from the second group differed in the content of the phosphoric, oxalic, pyrogallic, azelaic, 5-hydroxypipecolic acids; dulcitol; campesterol; undecyl and behenic fatty acids; and MAG-1 C18:1; while lower confidence levels (0.1 > p > 0.05) characterized the differences in the contents of 3-hydroxypipecolic acid, MAG-2 C18:3, and methyl-D-galactopyranoside. The third group was noted for the amounts of fumaric, pelargonic, pipecolic acids, ononitol, galactinol, and hydroquinone at p = 0.05, and for threonine, dulcitol, myoinositol, hydroxyoctacosenoic acid, and sucrose at 0.1 > p > 0.05.

A significant correlation was found between indicators of resistance to fungal pathogens and the group of nonproteinogenic amino acids, polyols, phytosterols, acylglycerols, mono- and oligosaccharides, glycosides, and phenolic compounds (hydroquinone, kempferol). No relationship was found between the sum of free amino acids and organic acids, on the one hand, and sensitivity to fungal pathogens, on the other hand.

The discriminant analysis of metabolomic data on the studied caryopses of Ae. tauschii accessions demonstrated separation of the latter into two main groups – resistant and susceptible to fungal pathogens – according to the canonical variable coefficient. The most “informationally important” characters that confirmed the difference in metabolite profiles and determined the separation of Ae. tauschii accessions between the group of resistant or susceptible ones with the 98 % accuracy were the levels of stigmasterol, MAG, DAG; of pelargonic, galacturonic, and 3-hydroxypipecolic acids; and of galactinol, glycerol, sorbose, maltose, tyrosine and glycosides. Among the above characters, stigmasterol and maltose values were the most statistically significant for the susceptible forms, while for the resistant ones it was DAG.

The “informationally important” characters and the classification function were used to analyze how the assumptions as for the resistance or susceptibility of Ae. tauschii accessions matched the reality. As a result, the status was confirmed for all the studied accessions, except for k-527, an Ae. tauschii accession from Armenia. The canonical discriminant analysis of metabolomic characters made it possible to clarify the differentiation parameters of resistant and susceptible forms of Ae. tauschii. The histogram of the canonical variable eigenvalues distribution according to their magnitude shows that the values for resistant accessions fall within the 0 to +5 range, while for the susceptible ones these values are found in the 0 to –5 range (see the Figure).

**Fig. 1.**
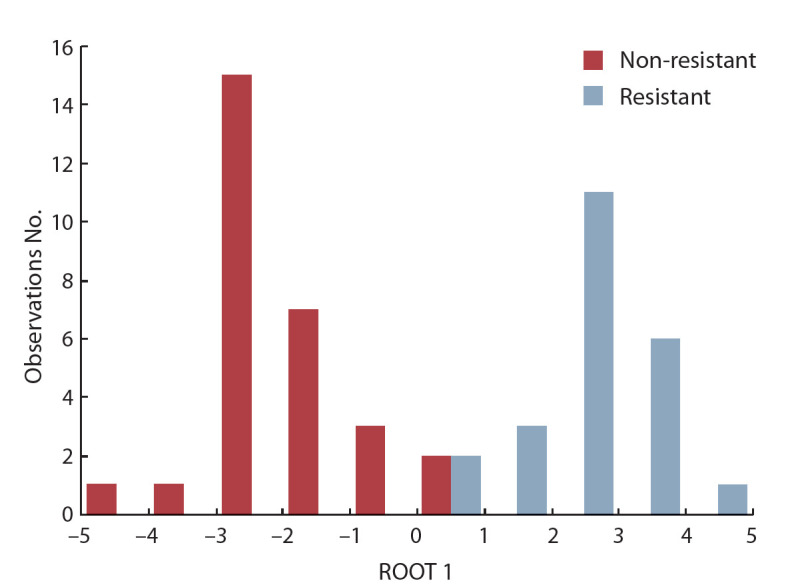
Histogram of the distribution of resistant and susceptible Ae. tauschii forms by the magnitude of the canonical variable eigenvalues.

## Discussion

The results of our work show that the metabolomic profiles of resistant and susceptible forms of Ae. tauschii differ significantly. When using the metabolomic analysis data, there is a high probability (up to 98 %) of identifying forms resistant to fungal pathogens among the accessions taken into the study without additional tests. We recommend that this approach be used to optimize the breeding process.

Analysis of metabolomic data for resistant and susceptible forms of Ae. taushii allows for a substantiated conclusion about the degree and nature of fungal pathogen influence on the main stages of primary and secondary metabolism in caryopses of accessions differing in the degree of resistance. To a greater or lesser degree, almost all pathogens affected metabolic processes, i. e., the Krebs cycle; glycolysis; and metabolism of fatty acids, acylglycerols, polyols, phytosterols, mono- and oligosaccharides. With all this, fungal pathogens had practically no effect on the contents of free amino acids (except for threonine and tyrosine). Since tyrosine is a precursor in the synthesis of many bioactive compounds that perform various functions – structural (lignin), protective (phenolic compounds, alkaloids, etc.), transport (electron transfer), and others – the effect of pathogens on the content of this amino acid in a caryopsis may be due to activation of defense mechanisms in response to the penetration of the pathogen into plant tissues, as confirmed by studies conducted outside Russia (Schenck, Maeda, 2018). Changes in the content of another amino acid, threonine, are associated by a number of authors with the influence of adverse biotic environmental factors, for example, insect pests. A plant reduces the concentration of substances necessary for the nutrition of the parasite, thereby affecting its population. The same mechanism may also act in cases of leaf rust and powdery mildew pathogens (Gonzales-Vigil et al., 2011). Azelaic acid, a product of oleic acid oxidation, and pipecolic acid (lysine catabolites) are intensely produced in response to the invasion of pathogens, in particular, fungi, into plant tissues; therefore, changes in the concentration of these compounds are well justified and confirmed by other researchers (Navarova et al., 2012; Zoeller et al., 2012).

Among free phenolic compounds, a significant effect on the resistance to fungal pathogens was detected for hydroquinone (the dominant compound of this group) and pyrogallic acid. Phenolic compounds are known to be actively involved in the formation of plant immunity. The presence of free forms of phenolic compounds most often indicates intensity of glycoside synthesis, where they function as aglycones. According to our data, mainly hydroquinone and pyrogallic acid accumulate in the caryopsis of resistant forms of Ae. tauschii. Their presence in the free state is most likely associated with the destruction of active forms, glycosides, exemplified by arbutin. As for pyrogallic acid, its accumulation may also be due to the plant/fungal pathogen interaction (the role of signaling substances) (Seigler, 1998; cit.: Gilbert, 2001).

The data from the present study confirm those we obtained earlier investigating the relationship between the resistance of oat (Avena sativa L.) forms to the Fusarium agent and such components of the metabolomic profile as acylglycerols, nonproteinogenic amino acids, and galactinol (Loskutov et al., 2019).

## Conclusion

The results of our study confirm the pertinence and effectiveness of the use of nonspecific metabolomic analysis for the search for and identification of plant forms with a set of compounds proposed as markers of resistance to certain pathogens. Ae. tauschii accessions with high contents of pipecolic acids, acylglycerols, galactinol, stigmasterol, maltose, tyrosine, sorbose, glycerol, azelaic and pyrogallic acids, methyl-Dgalactopyranoside, etc. can be included in breeding programs for cultivars of main cereal crops with high resistance to mildew, brown rust, and yellow rust.

## Conflict of interest

The authors declare no conflict of interest.
